# Seroma formation after parotidectomy: incidence, risk factors, and clinical implication

**DOI:** 10.3389/fsurg.2025.1704742

**Published:** 2026-01-26

**Authors:** Joo Hyun Kim

**Affiliations:** Department of Otorhinolaryngology-Head and Neck Surgery, Yongin Severance Hospital, Yonsei University College of Medicine, Gyeonggi-do, Republic of Korea

**Keywords:** parotidectomy, seroma, postoperative complications, risk factors, salivary gland neoplasms, drainage

## Abstract

**Background:**

Postoperative seroma is an underrecognized but clinically relevant complication after parotidectomy, leading to repeated aspirations and patient discomfort. Limited evidence exists on its incidence, risk factors, and clinical course.

**Methods:**

We retrospectively reviewed 527 patients who underwent parotidectomy between 2020 and 2025. Demographics, tumor characteristics, surgical details, and postoperative variables were analyzed. Seroma was defined as fluid accumulation requiring aspiration without evidence of salivary fistula. Persistent seroma was defined as requiring multiple aspirations or persisting beyond 14 days after drain removal. Multivariate logistic regression was performed to identify independent predictors, and subgroup analyses were conducted for persistent seroma.

**Results:**

Seroma occurred in 10.6% of patients. Independent risk factors included anterior tumor location (OR 2.21), larger tumor size (OR 1.58 per cm), body mass index ≥25 (OR 1.76), and use of facelift-type incisions (OR 1.92). Among patients with seroma, 33.9% developed persistent seroma, which was associated with larger tumors, higher BMI, greater aspirated volumes, and longer resolution times. Higher drain output prior to removal was also observed in the seroma group, suggesting that current thresholds for drain removal may be insufficient in high-risk patients.

**Conclusion:**

Anterior tumor location, larger tumor size, elevated body mass index, and facelift-type incisions are significant predictors of postoperative seroma after parotidectomy. Risk-adapted strategies, such as individualized drain removal protocols and early use of compression dressings, may reduce seroma-related morbidity.

## Introduction

Postoperative seroma is a relatively underrecognized but clinically significant complication following parotidectomy (1). It is defined as a localized collection of serous fluid predominantly lymphatic exudate and occasionally admixed with saliva within the surgical bed. Seroma typically develops after drain removal and may persist for several days to weeks. Although often self-limiting, it can lead to patient discomfort, cosmetic concerns, increased outpatient visits, and, in rare cases, secondary infection or wound dehiscence. The incidence of seroma after parotidectomy varies across studies, typically ranging from 3% to 10%, depending on surgical extent, technique, and postoperative care protocols (2, 3). While post-parotidectomy complications such as facial nerve injury, salivary fistula, Frey's syndrome, and hematoma have been widely reported, seroma formation has received comparatively less attention, despite its impact on patient recovery and follow-up care (4, 5). As a result, evidence-based strategies for preventing and managing seroma remain limited.

At our institution, parotidectomy is routinely performed for both benign and malignant tumors, using a spectrum of surgical techniques including extracapsular dissection (ECD), superficial, total, and robotic-assisted approaches. While most patients recover uneventfully, a subset develops persistent or recurrent seroma, requiring repeated needle aspirations or, in severe cases, vacuum-assisted drainage or secondary wound management. These cases represent not only a clinical problem but also a source of patient discomfort and increased healthcare utilization. To date, no validated risk model exists to predict seroma formation across diverse surgical settings. The present study aimed to evaluate the incidence and risk factors of postoperative seroma formation following parotidectomy in a broad clinical setting, encompassing different surgical techniques and tumor types. By identifying modifiable risk factors and evaluating potential preventive strategies, this study seeks to improve postoperative management and reduce the burden of this underrecognized complication.

## Methods

### Study design and patient selection

This retrospective cohort study was conducted at the Department of Otorhinolaryngology–Head and Neck Surgery, Yongin Severance Hospital, a high-volume tertiary center for salivary gland surgery. The study was approved by the Institutional Review Board of Yonsei University Health System, with a waiver of informed consent due to the retrospective nature of the analysis. We reviewed the medical records of all adult patients who underwent parotidectomy between March 2020 and March 2025. Both conventional and robotic approaches were included. Exclusion criteria were incomplete medical records, prior parotid surgery, and follow-up duration less than 30 days. To ensure a focused analysis of true postoperative seroma, patients who developed sialocele or salivary fistula were excluded. Sialocele was defined as a localized fluid collection that showed gustatory swelling or saliva-like aspirate with elevated amylase levels, while salivary fistula referred to persistent external drainage of amylase-rich fluid through the wound or drain site.

### Data collection and variables

Demographic and clinical variables were retrospectively collected from electronic medical records. These included patient age, sex, body mass index (BMI) and smoking status. Smoking status was categorized as current smoker, former smoker, or never smoker. Medical comorbidities with potential impact on wound healing, including diabetes mellitus, hypertension, and dyslipidemia, were also recorded based on preoperative medical evaluation. Tumor-related variables included histopathologic diagnosis, tumor size, and location. Tumor location was classified by lobe involvement (superficial vs. deep), based on operative findings and radiologic imaging, and by anatomical position (anterior vs. posterior). Anterior tumors were defined as those located anterior to a vertical line drawn through the lateral canthus and external auditory canal on axial magnetic resonance imaging (MRI) as previously described by Liu et al. (6). This landmark has been shown to correlate well with the radiologic and surgical anatomy of the parotid region and the course of the facial nerve. All patients underwent preoperative MRI to confirm tumor location and its relation to the facial nerve. Surgical variables included the type of parotidectomy performed, classified as ECD, partial superficial parotidectomy, superficial parotidectomy, or total parotidectomy. ECD was defined as the removal of the tumor with a narrow margin of surrounding tissues without intentional identification or dissection of the facial nerve. In contrast, partial superficial and superficial parotidectomy involved partial or complete resection of the superficial lobe with anatomical exposure of the facial nerve or its branches. The type of facial nerve dissection (anterograde or retrograde) was also recorded. An anterograde approach, starting from the main trunk toward the peripheral branches, was the standard technique, whereas a retrograde dissection, proceeding from the peripheral branches toward the main trunk, was selectively performed in cases of recurrent tumors or anterior-lobe lesions with limited main-trunk exposure. The surgical approach (conventional or robotic assisted), type of skin incision (modified Blair or modified facelift), and total operative time were documented. All surgeries were performed by a single experienced head and neck surgeon with extensive expertise in parotid gland procedures. In conventional parotidectomy cases, tissue dissection was primarily performed using cold instrument and the Thunderbeat® device (Olympus Corp., Tokyo, Japan), which combines ultrasonic and bipolar energy for simultaneous cutting and coagulation. Prominent arterial branches encountered during the procedure were controlled using surgical ties. In robotic-assisted surgeries, all procedures were performed using the da Vinci SP® Surgical System (Intuitive Surgical Inc., Sunnyvale, CA, USA). Dissection was performed using Maryland bipolar forceps and monopolar curved scissors, with energy delivered via the ERBE VIO 300D electrosurgical generator (ERBE Elektromedizin GmbH, Tübingen, Germany). For hemostasis of prominent arteries, endoscopic vascular clips were applied. Minor reconstructive procedures, such as SMAS advancement, temporoparietal fascia flap, or acellular dermal matrix (Megaderm®, L&C BIO Co., Ltd., Seongnam, Republic of Korea) placement, were selectively performed in patients with large parotid bed exposure to reduce contour depression and dead space. In all cases, a topical hemostatic agent was applied to the surgical bed at the conclusion of the procedure to promote hemostasis and minimize postoperative fluid accumulation. Drain removal was routinely performed when the 24-hour output fell below 20 mL, in accordance with institutional protocol, and patients were discharged shortly thereafter in the absence of complications. Patients were instructed to return to the hospital if they experienced symptoms suggestive of fluid accumulation at the surgical site after discharge. Postoperative management variables included the duration of surgical drain placement, total drain output before removal, and the use of facial compression bandages in the immediate postoperative period. In patients who developed seroma after discharge, additional variables were recorded, including the number of aspirations required, the total aspirated volume, and the time interval from drain removal to complete resolution of the seroma. Persistent seroma was defined as a collection requiring multiple aspirations or lasting beyond 14 days post-drain removal. Facial compression bandages were selectively applied in cases of evident postoperative swelling or when patients presented with suspected seroma formation after discharge.

### Statistical analysis

Continuous variables were summarized as means with standard deviations or medians with interquartile ranges, depending on data distribution. Categorical variables were presented as frequencies and percentages. Univariate analysis was performed using the Student's t-test or Mann–Whitney U test for continuous variables, and the chi-square test or Fisher's exact test for categorical variables. Variables with a *p*-value < 0.1 in univariate analyses or of known clinical relevance were entered into the multivariate logistic regression model to identify independent predictors of postoperative seroma. In addition, a subgroup analysis was conducted to investigate factors associated with persistent seroma, defined as fluid collection requiring repeated aspiration for more than 14 days after drain removal. The same univariate and multivariate procedures were applied to this subgroup. Model performance for predicting persistent seroma was evaluated using receiver operating characteristic (ROC) curve analysis, and the area under the curve (AUC) was reported. Statistical significance was defined as *p* < 0.05. All analyses were conducted using SPSS Statistics version 29.0 (IBM Corp., Armonk, NY, USA).

## Results

Of the 527 patients who underwent parotidectomy between March 2020 and March 2025, postoperative seroma was identified in 56 patients (10.6%). In addition to seroma, other salivary complications were recorded. Sialocele occurred in 7 patients (1.3%) and salivary fistula in 2 patients (0.4%). These cases were analyzed separately and were not included in the seroma group to ensure a focused assessment of true serous collections.

### Significant clinical and operative findings

The demographic, tumor-related, and surgical characteristics of patients with and without postoperative seroma are summarized in Table 1. Patients who developed seroma had a significantly higher mean BMI than those who did not (26.1 ± 3.4 vs. 24.1 ± 2.9; *p* = 0.038). When stratified by BMI, individuals with BMI ≥ 25 kg/m^2^ showed nearly double the incidence of seroma compared with those with BMI < 25 kg/m^2^ (14.2% vs. 7.1%; *p* = 0.021). The mean tumor size was also larger in the seroma group (3.7 ± 1.2 cm) than in the non-seroma group (2.2 ± 1.0 cm; *p* < 0.001). Anteriorly located tumors were more frequent among patients with seroma (71.4%, 40/56) than among those without (55.0%, 259/471; *p* = 0.041). Regarding incision type, the facelift-type incision was associated with a higher seroma rate than the modified Blair incision (64.3% vs. 48.4%; *p* = 0.034). The total drain output before removal was greater in the seroma group (105 ± 30 mL) than in the non-seroma group (92 ± 25 mL; *p* = 0.04).

**Table 1:** Demographic, tumor-related, and surgical characteristics of patients with and without postoperative seroma.

Variable	Total (*n* = 527)	Seroma (*n* = 56)	Non-seroma (*n* = 471)	*p*-value
Age (years), mean ± SD	53.6 ± 12.3	57.1 ± 12.0	53.2 ± 12.4	0.09
Sex (male), *n* (%)	339 (64.3%)	36 (65.2%)	303 (64.4%)	0.91
BMI, mean ± SD	24.3 ± 3.1	26.1 ± 3.4	24.1 ± 2.9	0.038
BMI ≥ 25, *n* (%)	237 (45.0%)	34 (60.7%)	203 (43.1%)	0.021
Smoking status, *n* (%)				0.29
Current smoker	78 (14.8%)	11 (19.6%)	67 (14.2%)	
Former smoker	132 (25.1%)	15 (27.5%)	117 (24.9%)	
Never smoker	317 (60.1%)	30 (52.9%)	287 (60.9%)	
Diabetes mellitus, *n* (%)	68 (12.9%)	8 (14.3%)	60 (12.7%)	0.62
Hypertension, *n* (%)	151 (28.7%)	17 (30.4%)	134 (28.5%)	0.73
Dyslipidemia, *n* (%)	96 (18.2%)	11 (19.6%)	85 (18.0%)	0.78
Tumor size (cm), mean ± SD	2.4 ± 1.1	3.7 ± 1.2	2.2 ± 1.0	< 0.001
Anterior tumor location, *n* (%)	299 (56.7%)	40 (71.4%)	259 (55.0%)	0.041
Tail portion only, *n* (%)	53 (10.1%)	5 (8.9%)	48 (10.2%)	0.74
Lobe involvement				0.12
Superficial	436 (82.8%)	48 (85.7%)	388 (82.4%)	
Deep	91 (17.3%)	8 (14.3%)	83 (17.6%)	
Histopathology				0.85
Pleomorphic adenoma	275 (52.2%)	32 (57.1%)	243 (51.6%)	
Warthin's tumor	143 (27.1%)	14 (25.0%)	129 (27.4%)	
Other benign tumors	72 (13.7%)	6 (10.7%)	66 (14.0%)	
Malignant tumors	37 (7%)	4 (7.1%)	33 (7.0%)	
Extent of surgery				0.63
Superficial	150 (28.5%)	20 (35.7%)	130 (27.6%)	
Partial superficial	173 (32.8%)	17 (30.4%)	156 (33.1%)	
ECD	159 (30.2%)	13 (23.2%)	146 (31.0%)	
Total	45 (8.5%)	6 (10.7%)	39 (8.3%)	
Facial nerve dissection				0.42
Anterograde	458 (86.9%)	48 (10.5%)	410 (89.5%)	
Retrograde	69 (13.1%)	8 (11.6%)	61 (88.4%)	
Surgical approach, *n* (%)				0.52
Conventional	433 (82.1%)	46 (82.1%)	387 (82.2%)	
Robotic-assisted	94 (17.9%)	10 (17.9%)	84 (17.8%)	

BMI, body mass index; ECD, extracapsular dissection.

### Other variables

Operative and postoperative management variables in patients with and without postoperative seroma are summarized in Table 2. No significant differences were observed between groups in terms of age (57.1 ± 12.0 vs. 53.2 ± 12.4 years; *p* = 0.18), sex (65.2% vs. 64.4% male; *p* = 0.91), or smoking status (*p* = 0.29). There were no significant differences in the prevalence of medical comorbidities, including diabetes mellitus, hypertension, and dyslipidemia, between the seroma and non-seroma groups (all *p* > 0.05). Tumor location in the tail or deep lobe, lobe involvement (superficial vs. deep; *p* = 0.12), and histopathologic type were not associated with seroma formation. Pleomorphic adenoma was the most frequent tumor in both groups, followed by Warthin's tumor (*p* = 0.85). The extent of parotidectomy (*p* = 0.63), surgical approach (robotic-assisted vs. conventional; *p* = 0.52), and facial-nerve dissection type (anterograde vs. retrograde; *p* = 0.42) likewise showed no significant association with seroma occurrence. An anterograde approach was performed in 458 patients (86.9%) and a retrograde approach in 69 patients (13.1%), the latter mainly for recurrent or anterior-lobe tumors with limited main-trunk exposure. Among the 527 patients, six (1.1%) underwent minor reconstruction of the parotid bed (SMAS advancement = 3, temporoparietal fascia fla*p* = 1, and Megaderm® acellular dermal matrix placement = 2). None of these patients developed postoperative seroma.

**Table 2:** Operative and postoperative management variables in patients with and without postoperative seroma.

Variable	Seroma (*n* = 56)	Non-seroma (*n* = 471)	*p*-value
Operative time (minutes), mean ± SD	121 ± 28	112 ± 26	0.08
Surgical approach			0.52
Robotic assisted	10 (17.9%)	71 (15.1%)	
Conventional	46 (82.1%)	400 (84.9%)	
Skin incision type			0.034
Facelift	36 (64.3%)	228 (48.4%)	
Blair	20 (35.7%)	243 (51.6%)	
Drain duration (days), mean ± SD	3.5 ± 1.0	3.2 ± 1.1	0.12
Total drain output (mL), mean ± SD	105 ± 30	92 ± 25	0.04
Compression bandage, *n* (%)	22 (39.3%)	41 (8.7%)	< 0.001

### Characteristics of seroma

**Figure 1:**
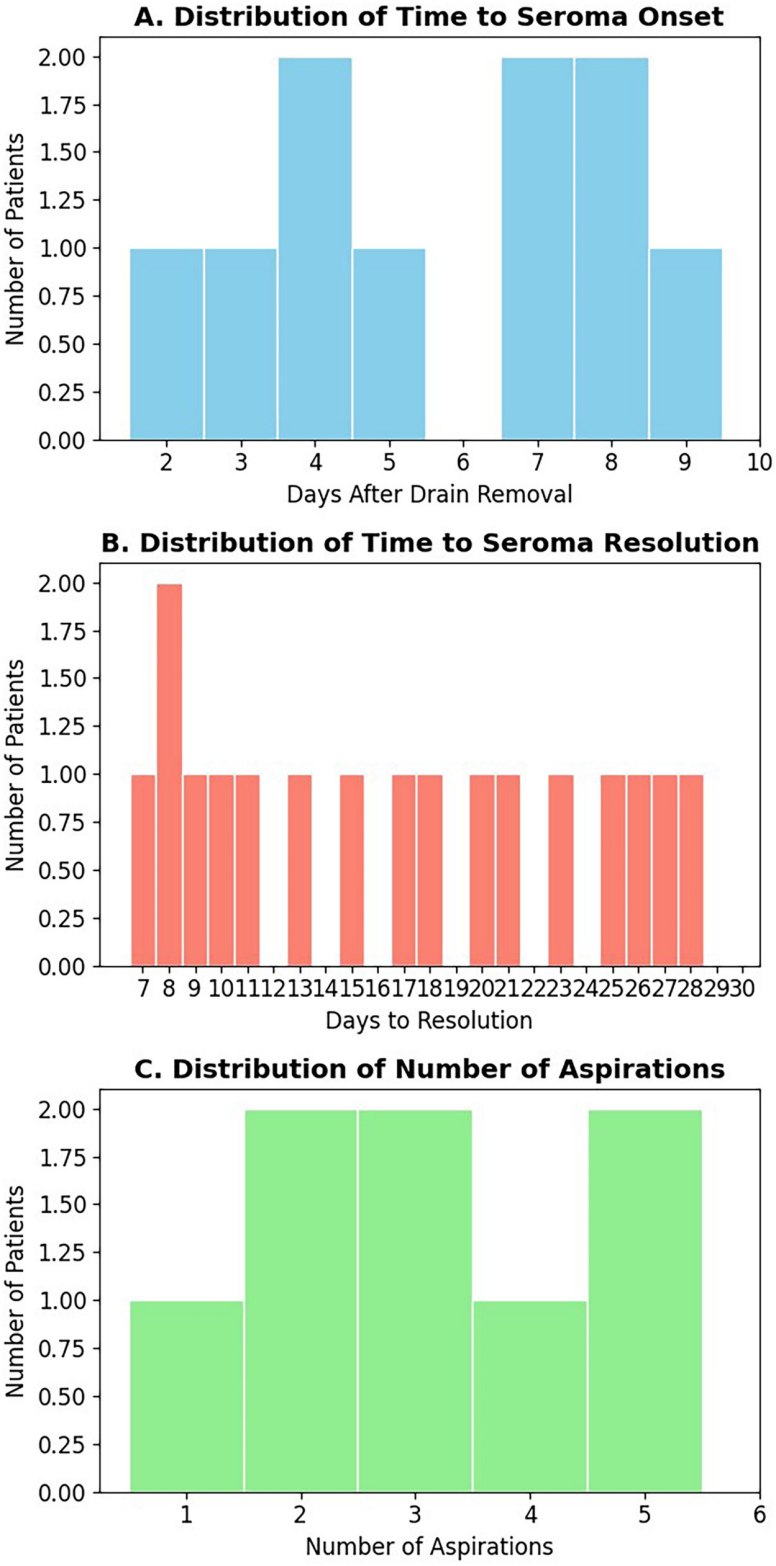
Distribution of time to seroma onset, number of aspirations, and duration to resolution among patients with postoperative seroma. **(A)** Time to initial seroma detection after drain removal. **(B)** Number of aspirations required for resolution. **(C)** Duration from onset to resolution. Data are shown as individual values with medians and interquartile ranges.

Among the 56 patients with seroma, fluid accumulation typically presented within the first postoperative week (median 4 days; range 2–10 days after drain removal). The median time to complete resolution was 17 days (range 7–31), requiring a median of 2 aspirations (range 1–6). The cumulative aspirated volume ranged from 30 to 240 mL (median 110 mL). Most cases resolved with intermittent aspiration and compression alone (Figure 1).

### Persistent seroma

Persistent seroma, defined as requiring multiple aspirations or lasting more than 14 days post-drain removal, was identified in 19 patients (33.9% of seroma cases; 3.6% of the total cohort). These patients had significantly larger tumors (4.0 ± 1.2 cm vs. 3.3 ± 1.1 cm; *p* = 0.03) and higher BMI (27.2 ± 2.8 vs. 25.4 ± 3.2; *p* = 0.048) than those with transient seroma. They also underwent more aspirations (median 3 vs. 1) and required a longer duration to resolution (median 24 vs. 12 days; *p* < 0.001) Compression bandages were more frequently applied in patients with persistent seroma (68.4%) compared to those with transient seroma (24.3%, *p* = 0.004), either prophylactically or as part of post-seroma management (Table 3).

**Table 3:** Comparison of clinical and management variables between persistent and transient postoperative seroma.

Variable	Persistent Seroma (*n* = 19)	Transient Seroma (*n* = 37)	*p*-value
Tumor size (cm), mean ± SD	4.0 ± 1.2	3.3 ± 1.1	0.03
BMI, mean ± SD	27.2 ± 2.8	25.4 ± 3.2	0.048
Number of aspirations, median (range)	3 (2–6)	1 (1–2)	< 0.001
Duration to resolution (days), median (range)	24 (15–31)	12 (7–18)	< 0.001
Total aspirated volume (mL), median (range)	150 (90–240)	90 (30–140)	0.017
Compression bandage *n* (%)	13 (68.4%)	9 (24.3%)	0.004

BMI, body mass index.

### Multivariate analysis

**Figure 2:**
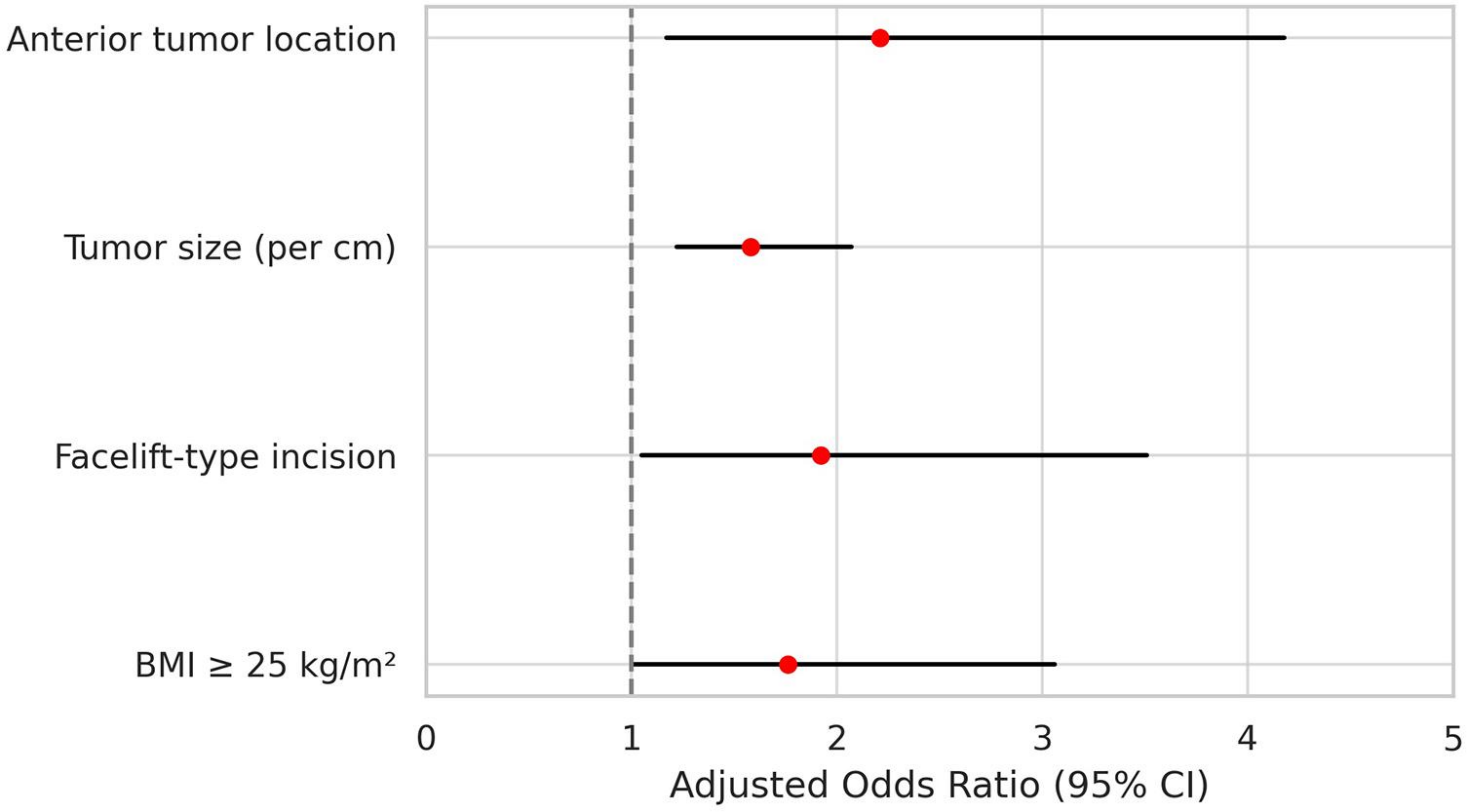
Forest plot of independent risk factors for postoperative seroma following parotidectomy. Forest plot showing adjusted odds ratios (ORs) with 95% confidence intervals (CIs) from multivariate logistic regression. Anterior tumor location, larger tumor size (per 1 cm), BMI ≥ 25 kg/m^2^, and facelift-type incision were associated with increased risk. The vertical line indicates OR = 1.

Multivariate logistic regression identified several independent predictors of postoperative seroma formation. Anterior tumor location was significantly associated with increased risk, with patients exhibiting more than twice the odds of seroma compared to those with posterior tumors (odds ratio [OR], 2.21; 95% confidence interval [CI], 1.17–4.18; *p* = 0.015). Tumor size was also a strong predictor; for each additional centimeter in diameter, the odds of developing seroma increased by 58% (OR, 1.58; 95% CI, 1.22–2.07; *p* < 0.001). Use of a facelift-type incision was associated with greater risk than the conventional modified Blair incision (OR, 1.92; 95% CI, 1.05–3.51; *p* = 0.034). In addition, a BMI of 25 kg/m^2^ or higher was independently associated with seroma formation (OR, 1.76; 95% CI, 1.01–3.06; *p* = 0.046). These results are summarized in Table 4, and a visual representation of the adjusted odds ratios with 95% confidence intervals is provided in Figure 2 as a forest plot.

**Table 4:** Multivariate logistic regression analysis of independent risk factors for postoperative seroma formation.

Variable	Adjusted OR	95% CI	*p*-value
Anterior tumor location	2.21	1.17–4.18	0.015
Tumor size (per 1 cm increase)	1.58	1.22–2.07	< 0.001
Facelift incision	1.92	1.05–3.51	0.034
BMI ≥ 25 kg/m^2^	1.76	1.01–3.06	0.046

BMI, body mass index; OR, odds ratio; CI, confidence interval.

## Discussion

Postoperative seroma is a relatively common complication following parotidectomy (1). Despite its frequency, the pathophysiologic mechanisms and consistent predictive factors remain incompletely understood, limiting the ability to implement effective preventive strategies. In this retrospective cohort of 527 patients, approximately 10% developed clinically significant seroma that required aspiration, with most cases occurring within the first postoperative week. Although seroma typically resolves with conservative treatment such as aspiration and compression dressings, a subset of patients experienced prolonged fluid accumulation lasting two to three weeks. In some cases, this was accompanied by localized inflammation or discomfort, highlighting the importance of early identification of high-risk patients and implementation of tailored postoperative management strategies.

Multivariate analysis revealed that anterior tumor location (OR 2.21), larger tumor size (OR 1.58 per cm), BMI ≥25 (OR 1.76), and the use of facelift-type incisions (OR 1.92) were independently associated with increased risk of postoperative seroma. Anteriorly located tumors showed a higher propensity for seroma formation than those located in the tail or deep lobe of the gland. This may be attributed to the wider surgical field and greater tissue mobilization required for anterior lesions, particularly in regions with limited fascial support and thinner subcutaneous tissues. Similarly, tumors arising from the superficial lobe were more frequently associated with seroma, although this did not reach statistical significance. Tumor size emerged as a strong independent predictor. The seroma group had significantly larger tumors than the non-seroma group (3.7 ± 1.2 cm vs. 2.2 ± 1.0 cm), and multivariate analysis confirmed a linear association between increasing size and seroma risk. This finding is consistent with the hypothesis that more extensive dissection and dead space creation promote postoperative fluid accumulation. Interestingly, patients with a BMI ≥ 25 kg/m^2^ were also more likely to develop seroma. Obesity has been previously implicated in seroma formation across various surgical fields, likely due to increased subcutaneous fat, impaired lymphatic drainage, and delayed wound healing (7–9). In the context of parotid surgery, larger gland size and thicker cervical soft tissue in obese individuals may similarly predispose to prolonged exudate accumulation.

Surgical approaches also influenced seroma risk. While robotic-assisted parotidectomy showed a trend toward lower seroma rates, this was not statistically significant. However, patients who underwent surgery via a facelift-type incision demonstrated significantly higher seroma incidence compared to those with a modified Blair incision. This may reflect a greater extent of subcutaneous flap elevation in facelift incisions, which can create larger potential spaces for fluid accumulation. The choice of incision may therefore have both cosmetic and functional implications, and careful consideration is warranted in high-risk cases.

Interestingly, the surgical extent including the use of extracapsular dissection did not significantly influence seroma formation, suggesting that the creation of dead space may be more affected by incision type and tumor characteristics than by the formal extent of parotidectomy. Likewise, the direction of facial nerve dissection (anterograde vs. retrograde) did not significantly affect seroma formation, indicating that the extent of flap elevation and the size of the dead space may play a more critical role than the dissection route itself. Furthermore, while Warthin tumors were relatively underrepresented in the seroma group, pleomorphic adenomas were the most common histology associated with seroma likely reflecting their prevalence and typical anterior location.

Drain duration and output offered further insights into seroma risk. Although the mean duration of drain placement was slightly shorter in the seroma group, their cumulative drain output prior to removal was significantly higher. This paradox suggests that removing drains solely based on daily output thresholds, such as 20 mL per day, may be insufficient in identifying residual fluid burden in high-risk patients. At our institution, drains were routinely removed when the 24-hour output fell below 20 mL. However, future protocols may benefit from incorporating patient-specific risk factors such as tumor size, location, BMI, or incision type into the decision-making process for drain removal.

Although the terms seroma and sialocele are sometimes used interchangeably in the context of post-parotidectomy fluid collections, they represent distinct clinical entities. In our cohort, sialocele and salivary fistula occurred in 1.3% and 0.4% of patients, respectively, and were analyzed separately to ensure a focused assessment of true seroma. Seroma was defined as an accumulation of lymphatic and interstitial fluid within the postoperative bed without gustatory swelling or biochemical evidence of salivary leakage, whereas sialocele was characterized by gustatory swelling or aspiration of amylase-rich fluid resulting from salivary duct disruption (10, 11).This distinction is clinically relevant, as management strategies differ: seromas often resolve with aspiration and compression, whereas sialocele may require prolonged drainage, anticholinergic agents, or revision surgery (12, 13).

Our findings suggest that seroma is more likely related to mechanical factors such as surgical dissection, dead space, and lymphatic disruption, rather than salivary leakage. This is supported by the observed associations with large tumor size, anterior location, and elevated BMI features known to increase tissue manipulation or impede fluid resorption. Furthermore, facelift-type incisions may enlarge the dissection plane and exacerbate postoperative fluid accumulation. Based on these observations, surgeons should consider risk-adapted strategies for high-risk patients. These may include extended drain retention, early application of compression dressings, and meticulous efforts to minimize dead space intraoperatively. Clear differentiation between seroma and sialocele not only guides appropriate postoperative management but also informs surgical planning to reduce preventable complications.

Previous work on post-parotidectomy fluid collections has primarily focused on sialocele rather than seroma, but the risk factors and mechanisms are overlapping. Witt reported the incidence and management patterns of sialocele after parotidectomy, emphasizing the role of dead space and ductal disruption as key contributors (14). Britt et al. further identified clinical factors associated with sialocele or salivary fistula formation, underscoring how greater tissue mobilization and ductal injury increase postoperative collections (10). In line with these observations, our data show that larger tumors and anterior location features that plausibly enlarge the dissected plane and disturb lymphatic/drainage pathways are independently associated with clinically significant seroma. Notably, although extent of surgery alone did not remain significant in multivariable analysis, factors that shape the geometry of the dissected bed (anterior topography and facelift-type flap elevation) were more strongly linked to seroma risk, suggesting that where and how the soft tissue is undermined may be more determinative than how much gland is removed.

Postoperative drainage management is also an important factor, as drainage strategy has been shown to influence fluid-related outcomes following superficial parotidectomy (15). Consistent with that perspective, our cohort showed higher cumulative drain output before removal in the seroma group despite similar drain duration, implying that a fixed daily threshold (< 20 mL/24 h) may underestimate residual burden in high-risk patients. Practically, patients with anteriorly located, larger tumors and elevated BMI could benefit from risk-adjusted protocols such as prolonged drainage, early and sustained compression, and meticulous dead-space control while recognizing that the choice of incision (facelift-type vs. modified Blair) may carry trade-offs between cosmesis and fluid accumulation. Prospective studies comparing standardized drain criteria and compression regimens across risk groups are warranted.

This study has several limitations. First, its retrospective design introduces inherent bias. Second, the clinical decision to aspirate and document seroma was at the surgeon's discretion, potentially underestimating minor or self-resolving cases. Finally, while we identified several independent risk factors, external validation in prospective cohorts is needed to refine prediction and prevention strategies.

## Conclusions

Anterior tumor location, large tumor size, elevated BMI, and use of facelift-type incisions are significant contributors to seroma formation after parotidectomy. Surgeons should be vigilant in identifying high-risk patients and consider tailored intraoperative and postoperative management including prolonged drain retention, pressure dressing, and dietary modifications to mitigate patient burden and improve outcomes.

## Data Availability

The raw data supporting the conclusions of this article will be made available by the authors, without undue reservation.
